# Comparative Outcomes of Mini Percutaneous Nephrolithotomy (Mini-PCNL) and Retrograde Intrarenal Surgery (RIRS) With Variable Laser Settings in the Management of 10–20 mm Lower Pole Renal Stones

**DOI:** 10.7759/cureus.90039

**Published:** 2025-08-13

**Authors:** Fatih Gokalp, Eser Ördek, Ekrem Yildirak, Hakan Sigva, Ömer Koraş, Ali Borekoglu, Sadik Gorur

**Affiliations:** 1 Department of Urology, Hatay Mustafa Kemal University, Faculty of Medicine, Hatay, TUR; 2 Department of Urology, Hatay Mustafa Kemal University, Hatay, TUR; 3 Department of Urology, Diyarbakir Gazi Yaşargil Education Research Hospital, Diyarbakir, TUR; 4 Deparment of Urology, Van Education and Research Hospital, Van, TUR; 5 Department of Urology, Izmir Tepecik Research and Education Hospital, İzmir, TUR; 6 Department of Urology, Mersin State Hospital, Mersin, TUR

**Keywords:** endourology, laser, lithotripsy, renal calculi, treatment of urolithiasis, urolithiasis

## Abstract

Objective: The treatment of urolithiasis is changing with the development of new technological tools and laser technologies. In this study, we aimed to compare the operation types and laser lithotripsy settings used in 1-2 cm lower calyceal stones and the effect of these settings on success.

Place & duration of study: Amasya University Faculty of Medicine, Şerefeddin Sabuncuoğlu Training and Research Hospital Department of Urology, Mustafa Kemal University Faculty of Medicine, between March 2019 and June 2021.

Methods: A total of 263 patients who underwent retrograde intrarenal surgery (RIRS) (n = 155) and mini percutaneous nephrolithotomy (mPCNL) (n = 108) for the treatment of lower pole kidney stones between 2019 and 2021 were analyzed. Laser settings between 0.2-0.8 joules & 10-20 Hz were defined as high frequency using low-pulse energy (HiFr-LoPE), and settings between 1-2 joules & 5-10 Hz were defined as low frequency using high-pulse energy (LoFr-HiPE). The operation types according to laser settings were compared in terms of operative time, fluoroscopy duration, length of hospital stay, stone clearance rate, clinically insignificant residual fragment (CIRF), and complication rate.

Results: The operation time and fluoroscopy time were significantly longer in the mPCNL group (p<0.001 and p<0.001, respectively). While the stone density was similar between groups (group 1 = 1039.00 (IQR = 930.00-1222.00) vs group 2 = 1101.00 (IQR = 900.00-1238.00); p = 0.406), the stone size was significantly higher in the mPCNL cohort (100.00 (IQR = 90.00 - 117.00) vs. 190.00 (IQR = 170.00-218.00), p<0.001). The stone-free rates (SFR) were similar between groups (p = 0.1011). Additionally, the required energy (J/mm^3^) was higher in the mPCNL group (23.70 (IQR=21.41-25.92) and 25.21 (IQR=24.29-26.19) p<0.001). For further analysis, subgroup analysis based on stone size 1.5 cm was performed and showed that there is no significant difference in terms of total energy, required energy, and stone-free rate between groups in patients with stone size below 1.5 cm (p = 0.589, p = 0.210, and p = 0.845, respectively). However, operation time, total energy, and required energy were significantly higher in the mPCNL group in patients with stone size above 1.5 cm (p <0.001, p <0.001, and p <0.001, respectively). The most common low-grade (grade I and grade II) complications were seen (22 and 8, respectively), and the grade of complications was similar between groups (p = 0.233).

Conclusion: The laser technologies have added a significant contribution to urinary stone treatment. The required energy and laser time may provide crucial information for preoperative planning. The bigger stones require greater energy for fragmentation, and the percutaneous approach is advantageous when planning the treatment of this stones.

## Introduction

The urolithiasis prevalence is increasing, and managing this evolution depends on the improvement of optical technology, ureteroscopy design, and novel laser technologies [1]. The choice of treatments, extracorporeal shockwave lithotripsy (ESWL), mini-percutaneous nephrolithotomy (mPCNL), and retrograde intrarenal surgery (RIRS), can be quite difficult in some patient groups, such as those with pelvis stones or lower calyx stones; especially for patients with a stone size between 10 and 20 mm, there is still a gray zone of treatment modalities. Percutaneous nephrolithotomy (PCNL) and contact lithotripsy are the major treatments with successful outcomes; however, higher complication rates, including hemorrhaging and the need for blood transfusions, still exist [2]. Miniaturizing the sheath size has reduced these complications, and mPCNL decreased complications compared to conventional PCNL [3]. Furthermore, in light of new technologies, RIRS has become a preferable treatment modality as an alternative to PCNL for stones smaller than 20 mm [4].

The widespread use of holmium laser in the management of urolithiasis has brought many discussions and debates [5]. The best laser setting and best fiber sizes for stone fragmentation are still controversial in the literature [6]. The laser settings are used by changing the two basic parameters: the pulse energy and pulse frequency [6,7]. There are currently no standard recommendations within mPCNL, although more specifically for the RIRS [8]. The fragmentation settings with miniaturization sheets increase operation time, and sometimes surgeons prefer bigger sheets for that reason. The literature has demonstrated that low frequency using high-pulse energy (LoFr-HiPE) settings has beneficial advantages when compared to high frequency using low-pulse energy (HiFr-LoPE) settings [9]. Higher density stones or bigger stone size. However, most of the studies were experimental [9,10]. The use of LoFr-HiPE for the treatment of large-sized (>2 cm) and high-density (>1000 HU) stones offers potential advantages over dusting in RIRS by enabling effective fragmentation and subsequent stone extraction. In the current study, we aimed to compare the clinical outcomes and procedural efficiency of PCNL and RIRS using variable laser settings in the treatment of 10-20 mm lower pole stones. The current study was presented as a meeting abstract at the 2022 7th National Congress of Minimally Invasive Urological Surgery, Antalya, Türkiye, March 10-13, 2022.

## Materials and methods

The patients who had mPCNL (n=108) and RIRS (n=155) for the management of lower pole kidney stones between 2020 and 2022 were examined after receiving local ethics committee approval. Patients under the age of 18, patients with a solitary kidney, or patients with multiple calyceal stones and malignancies were excluded from the study. All surgical options were decided upon after detailed discussion with the patients. mPCNL was planned and performed as the percutaneous surgical option.

A total of 213 patients were retrospectively analyzed. The demographic data of patients, in terms of side, size, density, operation time, fluoroscopy time, complication rates, and stone clearance rate (SCR), were recorded. All patients were evaluated preoperatively for the location and other properties of the stones. Stone volume (mm³) was calculated using software by automatic measurements after surgery. Before surgery, each patient’s urine culture was collected, and those who had an infection were put on the proper antibiotic regimen and underwent surgery once there was no growth in their urine culture. Two treatment types were assessed in terms of fluoroscopy time, operation time, hospital stay, SFR, and perioperative complications, and according to the Clavien-Dindo classification, postoperative complications were compared [11]. Operation time was determined to be the interval of time between the puncture with a needle into the collecting system and the skin closure in mPCNL, and the time duration should start from doing cystoscopy and introduction of guide wire and should end with completion of surgery in RIRS. Fluoroscopy time was defined as the duration of fluoroscopy usage, from skin puncture to the end of the procedure for PCNL and from ureteral access sheath (UAS) placement to the end of the procedure for RIRS. Laser time was defined as the time between the firing of the first and last laser pulses. Laser settings between 0.2-0.8 joules and 10-20 Hz were defined as high frequency using low-pulse energy (HiFr-LoPE), and settings between 1-2 joules & 5-10 Hz were defined as low frequency using high-pulse energy (LoFr-HiPE). The primary objective of our study was to compare the efficacy and safety of two distinct laser settings across two different treatment modalities (PCNL and RIRS). Our secondary outcome was the definition of the most appropriate laser setting depending on the characteristics of the stone.

Surgical procedures

The patients who had lower calyx stone size between 10 and 20 mm underwent mPCNL or RIRS. All operations were performed by four experienced senior surgeons. All RIRS operations were carried out on patients in the typical lithotomy position under general anesthesia. In accordance with the European Association of Urology (EAU) recommendations, all patients took antibiotic prophylaxis before the operation. Operations were performed with semirigid and flexible ureteroscopy, and a safety guidewire was inserted for all procedures. The UAS was placed at the beginning of the procedure. The stones were adequately dusted or fragmented using the holmium-YAG laser with a 200 nm laser fiber. A double-J stent was placed at the end of the operation according to the surgeon’s preference. All mPCNL procedures were performed in the standard prone position under general anesthesia. Patients were positioned in a prone position and provided with constructional silicone rolls. The radiographic contrast dye was injected through the ureteric catheter to visualize the collecting system, and under fluoroscopy, the system was punctured with a needle, and the urinary tract was dilated with Amplatz dilators by using a guidewire. A 16-20Fr sheath was placed, and a 15Fr-19Fr rigid nephroscope or 9.5Fr rigid ureteroscope was used. The stones were fragmented using the holmium:YAG laser (50 W) with a 200 nm laser fiber and were extracted using forceps or graspers. The nephrostomy tube was placed if needed, and the tubeless technique was preferred in cases that had no major bleeding or residual stones at the end of the procedure, a short operation time, or a single puncture. Operational data, including laser settings, total energy, fluid used for irrigation, and bleeding, were recorded. On the second postoperative day, the antegrade pyelography was performed, or the tube was clamped if there was no residual fragment or dye extravasation was not determined by imaging. The nephrostomy tube was removed, and patients were discharged after the drainage from the nephrostomy tract was stopped. If the drainage continued, we inserted a DJ stent. After the drainage was stopped, the patients were discharged.

Follow-up and statistical analysis

The first follow-up was done 4-6 weeks after discharge, and the visit included urinalysis and urinary ultrasonography. The SFRs were evaluated during the follow-up of the fourth week. The SFR was described as the absence of a residual stone or the absence of a fragment larger than two millimeters on tomography.

SPSS version 25 for Mac OS was used for statistical analysis. The compliance of continuous data to normal distribution was evaluated with the Shapiro-Wilk test. Continuous data were presented as median and interquartile range values. Categorical data were presented as numbers and percentages. Pearson’s chi-square or Fisher’s exact test was used in the comparison of categorical data according to their normality distribution. The Mann-Whitney U test was used for the comparison of non-normally distributed data. To evaluate the factors associated with SFRs and parameters, logistic regression analysis was performed. For further examination, the multivariate analysis was used for variables that were found to be significant at the univariate stage. A value of p <0.05 was considered statistically significant.

## Results

The median stone density and stone size were 1061 (925-1230) HU and 124 (96-180) mm². 16.2% of the patients had previous failed SWL histories. Intraoperative complications were seen in 9.9% of patients, and postoperative complications were seen in 15.6% of patients. The total SFR was 86.3% and reached up to 93.5% when clinically insignificant fragment cases were included (Table 1).

**Table 1:** Comparison of operation groups. ^€^Data was expressed as median and interquartile range. ^¥^Data was expressed as count and percentile. ^^^The Mann-Whitney U test was used. ^#^Fisher’s exact test was used. *Pearson chi-square test was used. w was the effect size Bold values were statistically significant. HU: Hounsfield unit; min: minute; kJ: kilojoules; HiFr-LoPE: High frequency - Low Power energy; LoFr-HiPE: Low frequency - High power energy. SF: stone-free; CSRF: clinically significant fragment; CIRF: clinically insignificant fragment

		RIRS Group (n=155)	mPCNL Group (n=108)	p-value
Age (years)^€^		57.00 (50.00-62.00)	56.00 (50.00-63.00)	0.819^
Body mass index^€^		25.55 (23.12-27.64)	25.45 (23.85-26.45)	0.940^
Gender^#^	Female	58 (37.4%)	40 (37.0%)	0.527*; w = 0.016
	Male	97 (62.6%)	68 (63.0%)	
History of extracorporeal shockwave lithotripsy^¥^	Absent	125 (80.6%)	46 (93.9%)	0.019#; w= 0.176
	Present	30 (19.4%)	3 (6.1%)	
History of operation^#^	Absent	117 (75.5%)	36 (73.5%)	0.456*; w = 0.072
	Present	38 (24.5%)	13 (26.5%)	
Stone density (HU)^€^		1039.00 (930.00-1222.00)	1101.00 (900.00-1238.00)	0.406^
Stone size (mm^2^)^€^		100.00 (90.00-117.00)	190.00 (170.00-218.00)	<0.001^
Intraoperative complication^¥^	Absent	140 (90.3%)	97 (89.8%)	0.276*; w = 9.675
	Malfunction	1 (0.6%)	1 (0.9%)	
	Bleeding	2 (1.3%)	2 (1.9%)	
	Perforation	6 (3.9%)	0 (0.0%)	
	Severally Bleeding	0 (0.0%)	1 (0.9%)	
	Not available	1 (0.6%)	3 (2.8%)	
	Mucosal Injury	4 (2.6%)	2 (1.9%)	
	Stent Migration	0 (0.0%)	0 (0.0%)	
	Steinstressia	1 (0.6%)	2 (1.9%)	
Operation time (min)^€^		50.00 (45.00-55.00)	78.00 (80.00-85.00)	<0.001^
Nephroscopy time (min)^€^		50.00 (45.00-55.00)	52.00 (45.00-57.00)	0.056^
Fluoroscopy time (min)^€^		14.00 (10.00-27.00)	42.00 (34.00-55.00)	<0.001^
Laser time (min)^€^		11.00 (10.00-12.00)	20.00 (18.00-23.00)	<0.001^
Total energy (kJ)^€^		9280.00 (7638.00-12003.00)	19729.00 (17724.00-23440.00)	<0.001^
Setting type^¥^	HiFr-LoPE	136 (87.7%)	10 (9.3%)	<0.001#
	LoFr-HiPE	19 (12.3%)	98 (90.7%)	
Stone-free rate^¥^	SF	139 (89.7%)	88 (81.5%)	0.101*; w = 0.171
	CSRF	9 (5.8%)	8 (7.4%)	
	CIRF	7 (4.5%)	12 (11.1%)	

The median age, gender, and history of operations were comparable between groups (p = 0.819, p = 0.527, and p = 0.456, respectively). While the stone density was similar between groups (p = 0.406), the stone size was significantly higher in the mPCNL cohort (100.00 (IQR = 90.00-117.00) vs. 190.00 (IQR = 170.00-218.00), p<0.001). The operation time and fluoroscopy time were shorter in the RIRS group (p<0.001 and p<0.001, respectively). The total laser time was significantly longer in the mPCNL group (11.00 min. (IQR = 10.00-12.00) vs. 20.00 min. (IQR = 18.00-23.00) p<0.001), and total laser energy usage was significantly higher in the MPCNL group (9280.00 joules (IQR = 7638.00-12003.00) vs. 19729.00 joules (IQR = 17724.00-23440.00) p<0.001). Additionally, the required energy (J/mm3) was higher in the mPCNL group (23.70 (IQR = 21.41-25.92) and 25.21 (IQR = 24.29-26.19), p<0.001). The SFRs were similar between groups (p = 0.101). Overall, the perioperative and postoperative complication rate was 9.7% and 13.5% in the RIRS group and 10.2% and 18.5% in the mPCNL group. The most common low-grade (grade I and grade II) complications were seen (22 and 8, respectively), and the grade of complications was similar between groups (p = 0.233) (Table 2). There was no significant difference between groups in terms of perioperative and postoperative complications (p = 0.276 and p = 0.178, respectively). No conversions occurred between RIRS and PCNL or the reverse.

**Table 2:** Comparison of postoperative complications. Data was expressed as count and percentile. *Pearson chi-square test was used. w was the effect size

		RIRS Group (n=155)	mPCNL Group (n=108)	p-value
Postoperative complication	Absent	132 (86.5%)	90 (81.5%)	0.178* w=0.087
	Present	23 (13.5%)	18 (18.5%)	
Postoperative grade of complication	Grade I	14 (9.0%)	8 (7.4%)	0.233* w=0.185
	Grade II	2 (1.3%)	6 (5.6%)	
	Grade IIIa	3 (1.9%)	3 (2.8%)	
	Grade IIIb	3 (1.9%)	1 (0.9%)	
	Grade IV	1 (0.6%)	0 (0.0%)	
	Grade V	0 (0.0%)	0 (0.0%)	

**Table 3:** Comparison of operation parameters depend on stone size *Data was expressed as median and interquartile range. ^#^Data was expressed as count and percentile. ^Mann-Whitney U test was used. ^#^Fisher’s Exact test was used. *Pearson Chi-square test was used. w was the effect size Bold values were statistically significant. HU: Hounsfield unit; min: minute; kJ: kilojoules; HiFr-LoPE: high frequency - low power energy; LoFr-HiPE: low frequency - high power energy.

		Size below 1.5 cm			Size above 1.5 cm		
		RIRS group (n=79)	mPCNL group (n=3)	p-value	RIRS group (n=76)	mPCNL group (n=105)	p-value
Stone density (HU)*		1102.00 (946.00-1219.00)	1102.00 (863.00-11637.00)	0.691	1004.00 (901.00-1227.00)	1100.00 (904.00-1240.00)	0.406
Stone size (mm^3^)*		420.00 (398.00-484.00)	496.00 (484.00-520.00)	0.063	363.00 (332.00-410.00)	794.00 (711.00-920.00)	<0.001^
Operation time (min)*		50.00 (45.00-55.00)	75.00 (75.00-85.00)	0.001^	45.00 (40.00-50.00)	80.00 (70.00-85.00)	<0.001^
Total energy (kJ)*		10947.00 (9137.00-12768.00)	13879.00 (11856.00-15689.00)	0.059	8137.50 (6961.00-9255.00)	19876.00 (17930.00-23867.00)	<0.001^
Laser time (min)*		32.00 (28.00-35.00)	32.00 (30.00-45.00)	0.589	29.50 (25.00-34.00)	38.00 (34.00-44.00)	<0.001^
Required energy (kJ/mm^3^)*		24.64 (22.73-26.53)	26.69 (23.90-32.42)	0.210	22.53 (21.18-24.50)	25.21 (24.33-26.16)	<0.001^
Setting type^#^	HiFr-LoPE	71 (89.9%)	2 (66.7%)	0.298; w = 0.142	65 (85.5%)	8 (7.6%)	<0.001^#^; w = 1.209
	LoFr-HiPE	8 (10.1%)	1 (33.3%)		11 (14.5%)	97 (92.4%)	
Stone-free rate^#^	SF	71 (89.8%)	3 (100%)	0.845; w = 0.065	68 (89.5%)	85 (81.1%)	0.181; w= 6.712
	CSRF	4 (5.1%)	0 (0.0%)		5 (6.6%)	8 (7.4%)	
	CIRF	4 (5.1%)	0 (0.0%)		3 (3.9%)	12 (11.4%)	

Further analysis, the subgroup analysis was done based on stone size below 1.5 cm and showed that there is no significant difference in terms of stone density, stone size, total energy, required energy, and SFR between groups in patients with stone size below 1.5 cm (p = 0.691, p = 0.063, p = 0.059, p = 0.589, p = 0.210, and p = 0.845, respectively) (Table 3). Furthermore, SFRs were similar between groups in patients with stone sizes above 1.5 cm (p = 0.181). However, stone size, operation time, total energy, and required energy were significantly higher in the MPCNL group in patients with stone sizes above 1.5 cm (p<0.001, p<0.001, p<0.001, and p<0.001, respectively).

Additional subgroup analysis in terms of laser setting showed that operation time was statistically higher in the LoFr-HiPE group (Table 4). However, the complication rate and SFR were similar between the different laser settings groups.

**Table 4:** Comparison of different laser settings’ group.

		LoPE-HiFr	HiPE-LoFr	p-value
Operation time		50.00 (45.00-55.00)	75.00 (65.00-85.00)	<0.001
Postoperative complication	Absent	122 (83.6%)	100 (83.6%)	0.251 x=6.727
	Grade I	16 (11.0%)	6 (5.1%)	
	Grade II	2 (1.4%)	6 (5.1%)	
	Grade IIIa	3 (2.1%)	3 (2.6%)	
	Grade IIIb	2 (1.4%)	2 (1.7%)	
	Grade IV	1 (0.7%)	0 (0.0%)	
	Grade V	0 (0.0%)	0 (0.0%)	
Sf status	SF	132 (90.4%)	95 (81.2%)	0.164 x=3.219
	CSRF	7 (4.8%)	10 (8.5%)	
	CIRF	7 (4.8%)	12 (10.3%)	

## Discussion

The treatment of lower pole stones between 10 and 20 mm in size could be challenging for urologists. Our study demonstrates that mPCNL has similar success rates with similar complications when compared with RIRS in patients with lower pole stones. Our study also demonstrated that while stone size increased, mPCNL with laser settings using LoFr-HiPE provided an advantage for treating lower pole stones.

Deciding the best management option for lower pole stones is still controversial. Lower pole stones could present significant difficulty during treatment due to calyx length, angling, and stone parameters [11,12]. A prospective study comparing SWL, RIRS, and PCNL for lower pole stones less than 20 mm in size showed that RIRS and PCNL had similar success rates (p = 0.092) with similar complication rates (p = 0.053), and the authors commented that even if stone retrieval was faster, the PCNL required a longer operation time [13]. The literature demonstrated that selecting the laser fragmentation type and retrieval method depends on stone density, volume, and location [14]. An experimental study showed that there is no difference in fragmentation volume when using different fiber sizes. However, the author commented that regardless of the pulse intensity, frequency, or total power applied, larger fibers were linked to broader fissures, and smaller fibers were linked to deeper fissures. In addition, fragmentation (HiPE-LoFr) settings provided benefits for bigger stones, with disadvantages such as the retropulsion of the stones [10]. Dusting (LoPE-HiFR) settings provided advantages with relatively small stones, such as shorter operation time and the avoidance of routine postoperative stenting due to smaller fragments being produced. The aforementioned results in the current literature suggest that total energy for stone fragmentation generally depends on stone location, size, and surgeon experience. Marques-Pinto et al.’s study showed an association between stone parameters and laser energy. However, there is no perfect correlation between total laser energy and operation time [17]. We think that this situation results from the fact that each laser pulse cannot make contact in such a way as to affect the stones, due especially to the surgeon's experience. In our work, all surgeons and staff were experienced surgeons in more than one hundred cases. In an early study, Sea et al. showed that fragmentation size was increased as pulse energy increased, and 2.0 J PE produced more than four times the fragmentation as 0.2 J PE with the same frequency settings [6]. The total energy needed for stone ablation is still unclear. A novel experimental study demonstrated that the mean required energy for calcium oxalate monohydrate (24±9 J/mm^3^) was significantly higher than those for cystine (7.6±3 J/mm^3^) and uric acid (2.5±0.7 J/mm^3^) (p<0.001) [16]. Furthermore, Marques‐Pinto et al.’s study investigated the relation between stone properties and laser energy parameters in RIRS, demonstrating that the median 22.3 kJ/cm3 (13.4-36.0) energy needed for the fragmentation of stone does not depend on stone composition. The authors of this study also commented that denser, larger stones require less lithotripsy and are associated with higher total laser energy [15]. Similar to findings in the literature, our study showed that the required energy (23.70 J/mm^3^ for RIRS and 25.21 J/mm^3^ for mPCNL) was similar for stone fragmentation and that the laser time was significantly higher in the MPCNL group.

The main goal of stone-removal surgery is to achieve complete SFR with low complications [11,17]. The literature demonstrates that, compared to mPCNL, RIRS delivers the best outcomes in terms of operation time, fluoroscopy time, and hospital stay [13]. However, a novel systematic review and meta-analysis comparing mPCNL and RIRS for 10-20 mm lower pole stones showed that mPCNL has higher SFRs than RIRS (p = 0.005) [11]. Controversial for this review, Shabana et al.’s study, which used a regression model, propensity score matching that creates similar patient groups for proper comparison, showed that SFRs were not higher in the mPCNL group than in the RIRS group when patients were categorized with PSM [14]. Our study demonstrated similar results: SFRs were similar between the mPCNL and RIRS groups, with larger stone sizes in the mPCNL group. However, total laser energy and required energy were significantly higher in the mPCNL group. We think that our study also revealed that the similar success rates in different stone volumes also depended on using HiPE-LoFr laser settings for stone fragmentation. Additionally, operation time was higher in the HiPE-LoFr group. We think that these findings are a result of the higher stone size in the LoPE-HiFr group.

Complications, as well as the SFR, are taken into consideration when deciding on the treatment of lower pole stones. PCNL traditionally has higher complication rates than other minimally invasive treatment modalities [18,19]. A meta-analysis and systematic review comparing the efficacy of conventional PCNL, RIRS, and SWL in patients with lower pole stones demonstrated that there is no significant difference in terms of complication rate between the three operation techniques (RIRS, OR = 1.42, 95% CI:0.91-2.21, p = 0.12, I2 = 0%) and ESWL, OR = 0.41, 95% CI: 0.16-1.09, p = 0.07, I2 = 70%) [12]. mPCNL involves miniaturized tools and fewer complications with similar SFRs compared to conventional PCNL. A systematic review and meta-analysis that compared the safety and efficacy of mPCNL and RIRS demonstrated that the overall complication rate was not different between two operation types (n = 171/877, 19.5 ± 19.1% vs. n = 112/721, 15.5 ± 18.9%, respectively (OR 1.43; 95% CI: 0.85-2.4, p = 0.18) [20]. As with this review, Shabana et al.’s study also showed that there were no significant complications noted between groups [14]. Our study showed similar results: that there was no significant difference between groups in terms of the Stava and Clavien classifications for grading of complications between the RIRS and mPCNL groups.

Our study has some inherent limitations. The primary limitation of this study is its retrospective design, which may introduce selection and reporting biases; moreover, the absence of randomization and strict procedural standardization across all cases limits the strength of causal inferences. Additionally, detailed anatomical parameters of the lower pole, such as infundibular length and infundibulopelvic angle, were not consistently available and thus could not be included in the analysis, although these factors are known to influence SCRs. Future prospective randomized studies with predefined surgical protocols are needed to validate our findings. Second, we only assessed lower pole stones and did not carry out a chemical analysis of the stones. Third, the total energy was noted at the end of the procedure. However, laser lithotripsy efficacy depends on equipment and surgeon experience, and for this reason, multicenter studies with much larger numbers of patients must be planned for future studies. Another important limitation was the use of the short-pulse holmium:YAG laser, which is commonly used in clinics; however, the thulium laser also provides advantages for stone-removal surgery. Additionally, novel comparisons could be planned with the new modalities and long pulse energy.

## Conclusions

Laser technologies have an important place in the treatment of urinary system stone disease and have already become an indispensable tool despite new modalities. Stone characteristics determine the laser settings needed for treatment. While bigger and denser stones will require high-pulse energy and low frequency for fragmentation before extraction through a percutaneous approach, smaller stones, on the other hand, could be dusted through RIRS and washed out through the ureter with low-pulse energy and high-frequency settings.
